# Changes in Gut Microbiota Composition Associated with the Presence of Enteric Protist *Blastocystis* in Captive Forest Musk Deer (*Moschus Berezovskii*)

**DOI:** 10.1128/spectrum.02269-21

**Published:** 2022-06-23

**Authors:** Lei Deng, Shanyu Chen, Wanyu Meng, Ziyao Zhou, Haifeng Liu, Zhijun Zhong, Hualin Fu, Liuhong Shen, Suizhong Cao, Kevin S. W. Tan, Guangneng Peng

**Affiliations:** a The Key Laboratory of Animal Disease and Human Health of Sichuan Province, College of Veterinary Medicine, Sichuan Agricultural University, Chengdu, China; b Laboratory of Molecular and Cellular Parasitology, Healthy Longevity Translational Research Programme and Department of Microbiology and Immunology, Yong Loo Lin School of Medicine, National University of Singapore, Singapore, Singapore; University of Georgia

**Keywords:** *Blastocystis*, captive management, conservation, forest musk deer, gut microbiome, wildlife disease

## Abstract

*Blastocystis* is a common protistan parasite inhabiting the gastrointestinal tract of a wide range of hosts including humans and domestic and wild animals. Many studies have revealed the associations between *Blastocystis* and gut microbiome in humans. However, only a few studies have focused on the associations between *Blastocystis* and gut microbiome of animals, especially in forest musk deer (*Moschus berezovskii*). We investigated the effects of the *Blastocystis* colonization on the intestinal bacterial community compositions using amplicon sequencing targeting the V4 variable region of the 16S rRNA. Two subtypes of *Blastocystis* (ST5 and ST10) and *Blastocystis*-free (control) were included in this study. We found that compared with the forest musk deer without *Blastocystis*, ST10-colonized forest musk deer had higher bacterial richness and diversity, while ST5-colonized forest musk deer showed a comparable bacterial diversity. Likewise, beta diversity revealed significant differences in bacterial community structure between ST10-colonized and *Blastocystis*-free forest musk deer. The proportion of *Bacteroidetes* were significantly enriched in ST10-colonized forest musk deer. Bacterial community structure between ST5-colonized and *Blastocystis*-free forest musk deer did not differ significantly. The present study explored the associations between *Blastocystis* and gut microbial community of forest musk deer for the first time, and revealed ST10 colonization, instead of ST5, is associated with higher bacterial diversity and shifted microbial structure. Our data provides valuable insights into the associations between gut microbiomes and parasites.

**IMPORTANCE** Forest musk deer is listed as an endangered species by International Union for Conservation of Nature Red List, and the Chinese government has introduced captivity breeding measures to curb the rapid decline of the musk deer population since the 1950s. It has been suggested that *Blastocystis* colonization can modulate the composition of the host's intestinal microbiota, thereby affecting the host health. The present study investigated the effects of the *Blastocystis* colonization on the gut microbiota in the feces of forest musk deer in Sichuan Province, China. Two subtypes (ST5 and ST10) have differential effects on the bacterial diversity and community composition, suggesting that the study of *Blastocystis* should be distinguished at the subtype level. Because the pathogenicity of *Blastocystis* is controversial, pathogenic, or commensal, continuous monitoring of the impact of *Blastocystis* colonization on the intestinal microbiota is of great significance to assess its health effects on forest musk deer.

## INTRODUCTION

The gastrointestinal tract is a dynamic and diverse ecosystem composed of trillions of bacteria, archaea, viruses, and protozoa (1). The evolution of the host and gut microbiota are closely related over millions of years (2). Studies have demonstrated a largely mutualistic relationship between the host and its gut microbiota, which play a crucial role in maintaining immune homeostasis, promoting host energy metabolism, and limiting pathogens invasion (3). Tremendous amount of data have revealed the relationships between gut bacterial community and host health and diseases (4–6), but interest in the study of other types of microorganisms, such as protozoan protists, has been rising over the past decade (7). Most of the protozoan protists have long been considered parasites and are thought to have adverse effects on the host (8). However, emerging evidence suggests that many common eukaryotes in the human gut, such as *Blastocystis*, are commensal (i.e., benefiting from interacting with the host without affecting it) or beneficial rather than pathogenic (9, 10).

*Blastocystis* is a common protist that commonly resides in the intestinal tract of humans and various animals, with a widespread distribution (11). *Blastocystis* has been identified as a member of the stramenopile phylum, a complex and heterogeneous evolutionary assemblage of heterotrophic and photosynthetic protists (12). The pathogenicity of *Blastocystis* is still controversial, there are studies associating it with symptoms of a variety of gastrointestinal disorders such as irritable bowel syndrome (IBS) and inflammatory bowel disease (IBD) (13–15). However, other studies have revealed *Blastocystis* as a common commensal or even a beneficial member of the human gut microbiome (10, 16, 17). Based on the analysis of small subunit ribosomal DNA (*SSU* rRNA) gene, 25 different subtypes have been identified in humans and a wide range of animals (18). Different *Blastocystis* subtypes exhibited different pathogenicity, growth rates, drug susceptibilities, host ranges, and other biological features (19, 20).

*Blastocystis* has been previously identified in a variety of captive wildlife in southwestern of China and can be maintained and transmitted among different wildlife (21). The forest musk deer (*Moschus berezovskii*), listed as an endangered species by International Union for Conservation of Nature (IUCN) Red List (22), is a small ruminant widely distributed in Central to Southern China (23). Its population has declined sharply in the past few decades due to habitat destruction, overexploitation, and illegal hunting (24). The Chinese government launched the artificial breeding of forest musk deer program in the 1950s to curb the rapid decline of the forest musk deer population (25). Although captive breeding of forest musk deer population in China has achieved some progress, gastrointestinal diseases are still an important factor affecting the population growth (26). Some studies have reported that forest musk deer are infected with parasites (27, 28). However, there are no reports on *Blastocystis* colonization in forest musk deer, and its association with gut microbiota has not been explored.

Herein we investigated the influence of *Blastocystis* colonization on gut bacterial communities in captive forest musk deer and compared the associations of different subtypes on the intestinal bacterial community. The gut microbial community was characterized by V4 region of the 16S rRNA gene (Illumina MiSeq) sequencing and bioinformatics analysis on two subtypes (ST5 and ST10) and *Blastocystis*-free forest musk deer. This study is the first to investigate the association between *Blastocystis* and bacterial communities in the gut of captive forest musk deer.

## RESULTS

### Assessment of sequence data.

A total of 989,204 reads were obtained from 16S rRNA amplicon sequencing. After quality filtering and processing, the total read count was measured at 964,004 with an average of 33,242 reads per sample. The minimum and maximum counts per sample were 29,428 and 39,018 respectively. As the sequencing depth increases, the rarefaction curves of observed amplicon sequence variants (ASVs) gradually become flat (around 6,000 reads), demonstrating that a sufficient sequencing depth was obtained to reflect the maximum level of bacterial diversity (Fig. S1 in the supplemental material).

### Alpha diversity analysis of gut microbiota in *Blastocystis*-colonized and *Blastocystis*-free forest musk deer.

**FIG 1:**
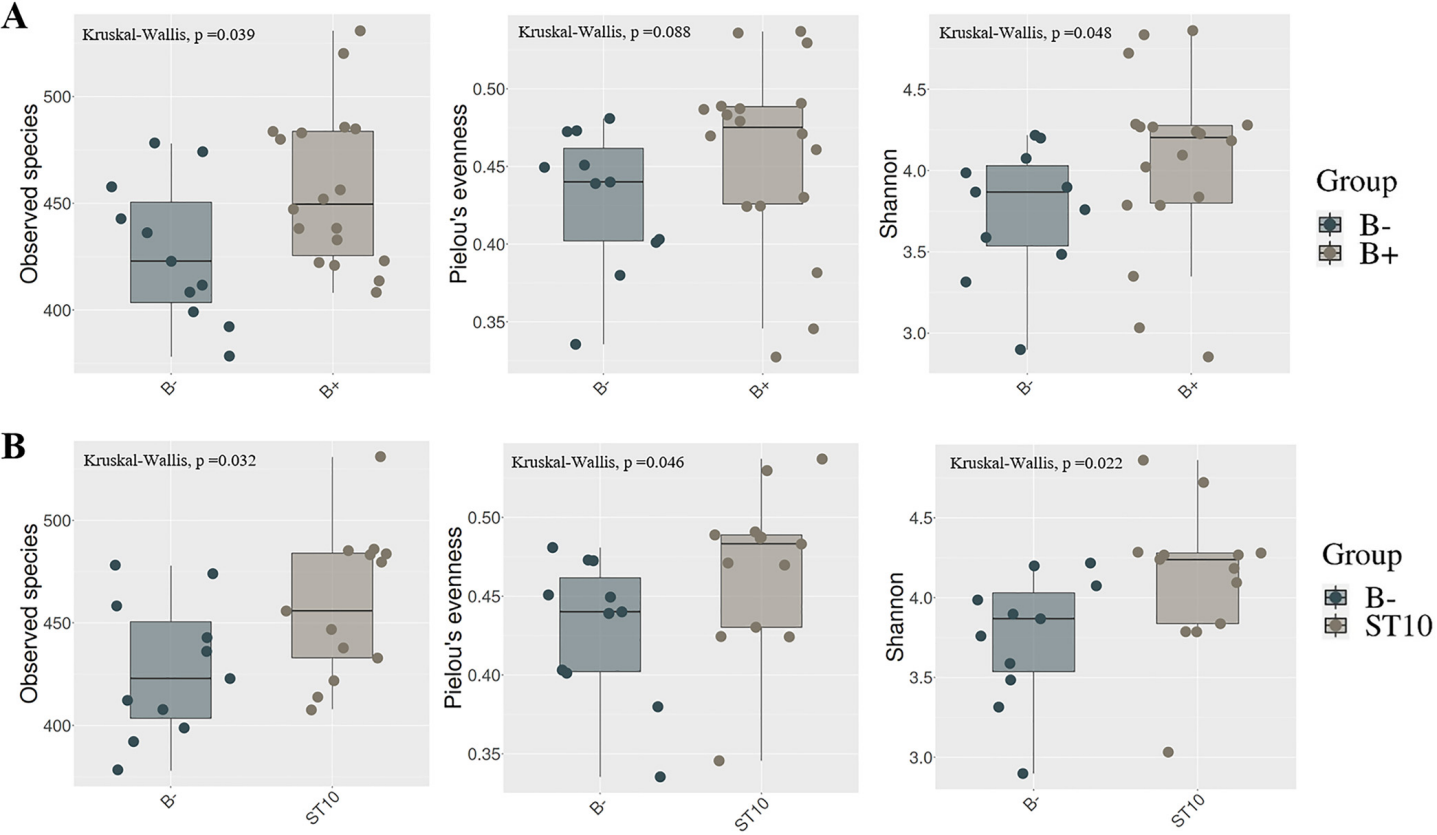
Comparison of alpha diversity of gut microbiota from the *Blastocystis*-colonized and *Blastocystis*-free groups based on Observed species, Pielou's evenness, and Shannon indexes (A). Comparison of alpha diversity of gut microbiota from the *Blastocystis*-free, and ST10-colonzed groups (B). B-: *Blastocystis*-free forest musk deer; ST10: ST10-colonized forest musk deer.

Alpha diversity was measured by Observed species, Pielou's evenness, and Shannon indexes. Observed species index was used to estimate the ASV number of the samples, reflecting the bacterial richness in the fecal samples of forest musk deer. Pielou's evenness index mostly focuses on the equitability between species. Shannon index takes into account the number of species and the proportion of each species within the community. Although there is no significant difference in Pielou's evenness index (Kruskal-Wallis test; *H *=* *61, *df *=* *1, *P = *0.088) between *Blastocystis*-colonized and *Blastocystis*-free groups, we observed significant difference in terms of observed species (*H *=* *53, *df *=* *1, *P = *0.039), and Shannon index (*H *=* *55, *df *=* *1 *P = *0.048) between the two groups (Fig. 1A). Due to *Blastocystis* comprising more than 25 distinct subtypes, possibly different species, and different effects on host immunity and gut microbiota having been observed, it is reasonable to study the effects of *Blastocystis* on gut microbiome at the subtype level (29). We thus further compared the fecal bacterial diversity of forest musk deer at the *Blastocystis* subtype level. Interestingly, we observed significant higher bacterial richness and diversity, measured by Observed species (*H *=* *34.5, *df *=* *1, *P = *0.032), Pielou's evenness index (*H *=* *37, *df *=* *1, *P = *0.046), and Shannon index (*H *=* *32, *df *=* *1, *P = *0.022) respectively, in ST10-colonized forest musk deer than those in *Blastocystis*-free forest musk deer (Fig. 1B), while ST5-colonized forest musk deer did not show this similar trend (all *P > *0.05, Fig. S2 in the supplemental material). Collectively, these data point toward a greater microbial diversity in the fecal microbiota of ST10-colonized forest musk deer, but not in ST5-colonized forest musk deer.

### Beta-diversity analysis of gut microbiota in *Blastocystis*-colonized and *Blastocystis*-free forest musk deer.

**FIG 2:**
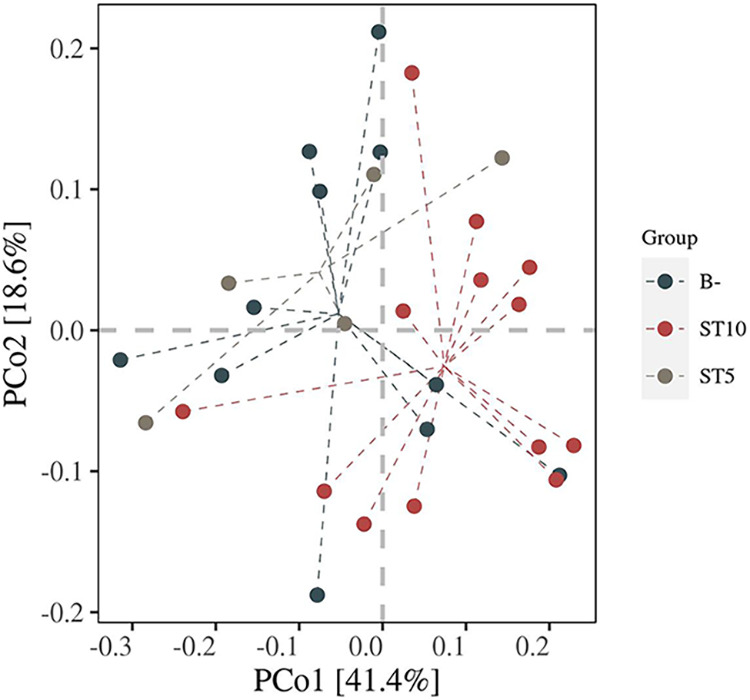
Beta-diversity analysis using PCoA based on Weighted Unifrac distance. Each point in the figure represents a sample, and points of different colors indicate different groups.

UniFrac, a distance metric, is widely used for comparing microbial community structures among different groups, including unweighted UniFrac and weighted UniFrac methods. The former only considers the presence or absence of observed species, while the latter, which we have chosen to adopt, accounts for the abundance of observed species. There were no significant differences in bacterial microbial communities between sampling locations (PERMANOVA, *R*^2^ = 0.06, *df *=* *1, *P = *0.096). Interestingly, the microbial community structures between ST10-colonized and *Blastocystis*-free forest musk deer were significantly different (*R*^2^ = 0.09, *df *=* *1, *P = *0.0039, Fig. 2), suggesting *Blastocystis* ST10 colonization induced significant shifts in the fecal bacterial community composition. In contrast, there was no significant difference in community composition between ST5-colonoized and *Blastocystis*-free forest musk deer (*R*^2^ = 0.03, *df *=* *1, *P = *0.914, Fig. 2).

### Comparison of the gut microbiota between *Blastocystis*-colonized and *Blastocystis*-free forest musk deer.

**FIG 3:**
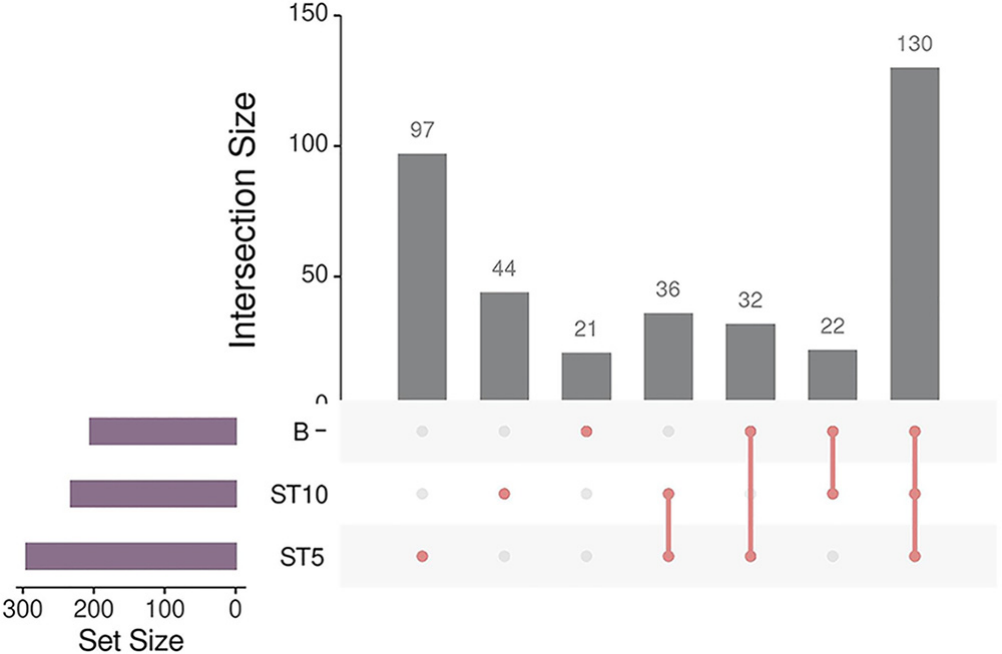
UpSet plot shows the numbers of ASVs that were shared or not shared by *Blastocystis*-free, ST10-colonized, ST5-colonized forest musk deer.

A total of 130 bacterial communities were identified in all groups, and 36 genera were found both in ST10 and ST5-colonized forest muck deer (Fig. 3). In addition, there are 32 and 22 genera shared by ST5-colonized and *Blastocystis*-free individuals, and ST10-colonized and *Blastocystis*-free forest muck deer, respectively (Fig. 3).

### Taxonomic composition of *Blastocystis*-colonized and *Blastocystis*-free forest musk deer gut microbiome.

**FIG 4:**
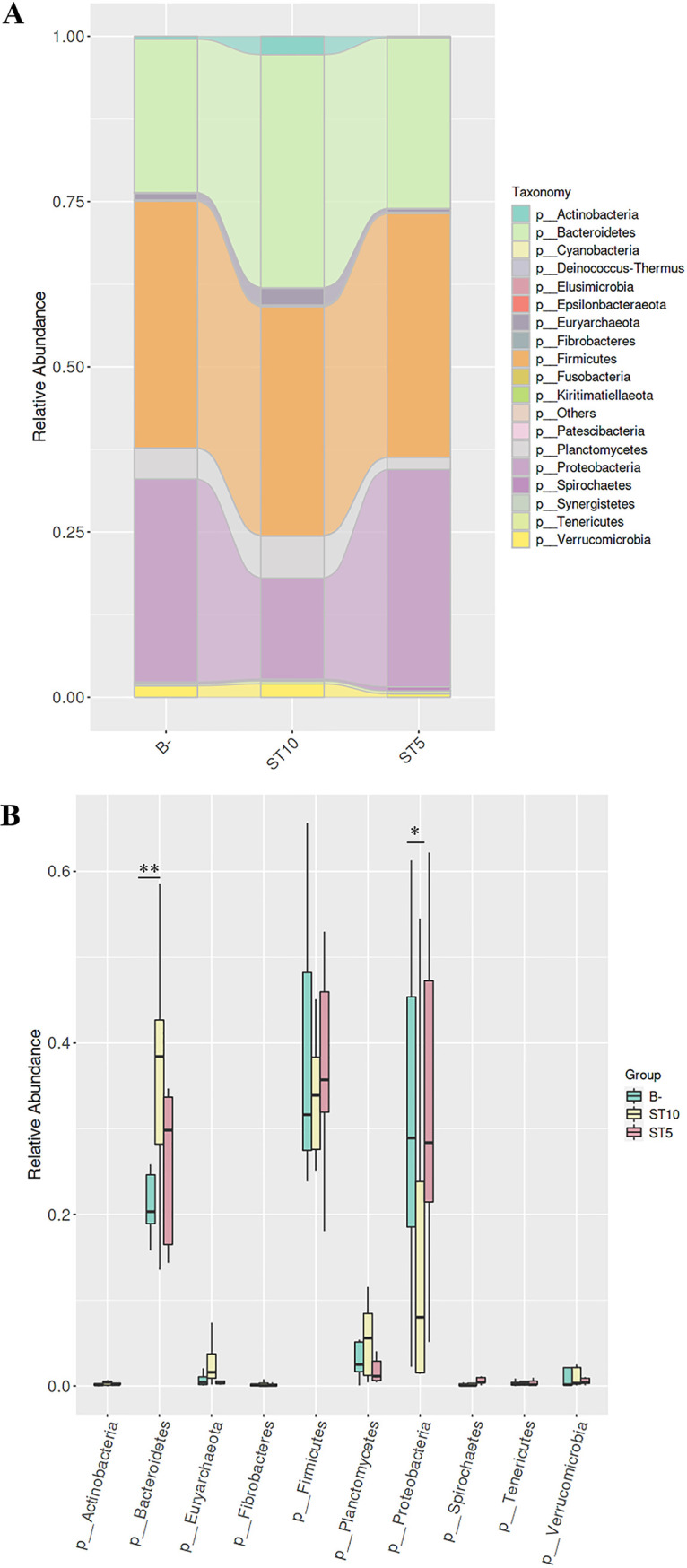
Relative abundance of the 19 phyla (A). Relative abundance (mean ± SD) of 10 major bacterial phyla (B) *Blastocystis*-free, ST10-colonized, and ST5-colonized forest musk deer. Wilcoxon rank-sum test. *, *P < *0.05, * *, *P < *0.01.

Sequence analysis showed all the ASVs from *Blastocystis*-colonized and *Blastocystis*-free forest musk deer were classified into 18 phyla, 24 classes, 41 orders, 68 families, and 190 genera. At the phylum level, Sankey diagram showed the relative abundances at the 19 bacterial communities among three groups (Fig. 4A). *Firmicutes* (36.3% ± 11%) and *Bacteroidetes* (28.1 ± 9.9%) dominated, followed by *Proteobacteria* (26.3% ± 17.7%) in the three groups (Fig. 4A). In ST10-colonized group, the proportion of *Bacteroidetes* was significantly higher than that in *Blastocystis*-free forest musk deer (Wilcoxon rank-sum test; *W *=* *27, *df *=* *1, *P = *0.0088) (Fig. 4B). In contrast, the level of the *Proteobacteria* in ST10-colonized forest musk deer was significantly lower than that in *Blastocystis*-free forest musk deer (*W *=* *34, *df *=* *1, *P = *0.03) (Fig. 4B). There was no significant difference of the top 10 bacterial communities in ST5-colonized and *Blastocystis*-free forest musk deer (all *P > *0.05, Fig. 4B). Taxonomic composition at the family level was also determined in the three groups (Fig. S3). Likewise, the family of *Bacteroidaceae* and *Rikenellaceae*, belonging to *Bacteroidetes*, were enriched in ST10-colonized forest musk deer (all *P < *0.05, Fig. S3 in the supplemental material)., whereas there was no significant difference in ST5-colonized and *Blastocystis*-free forest musk deer (all *P > *0.05, Fig. S3).

**FIG 5:**
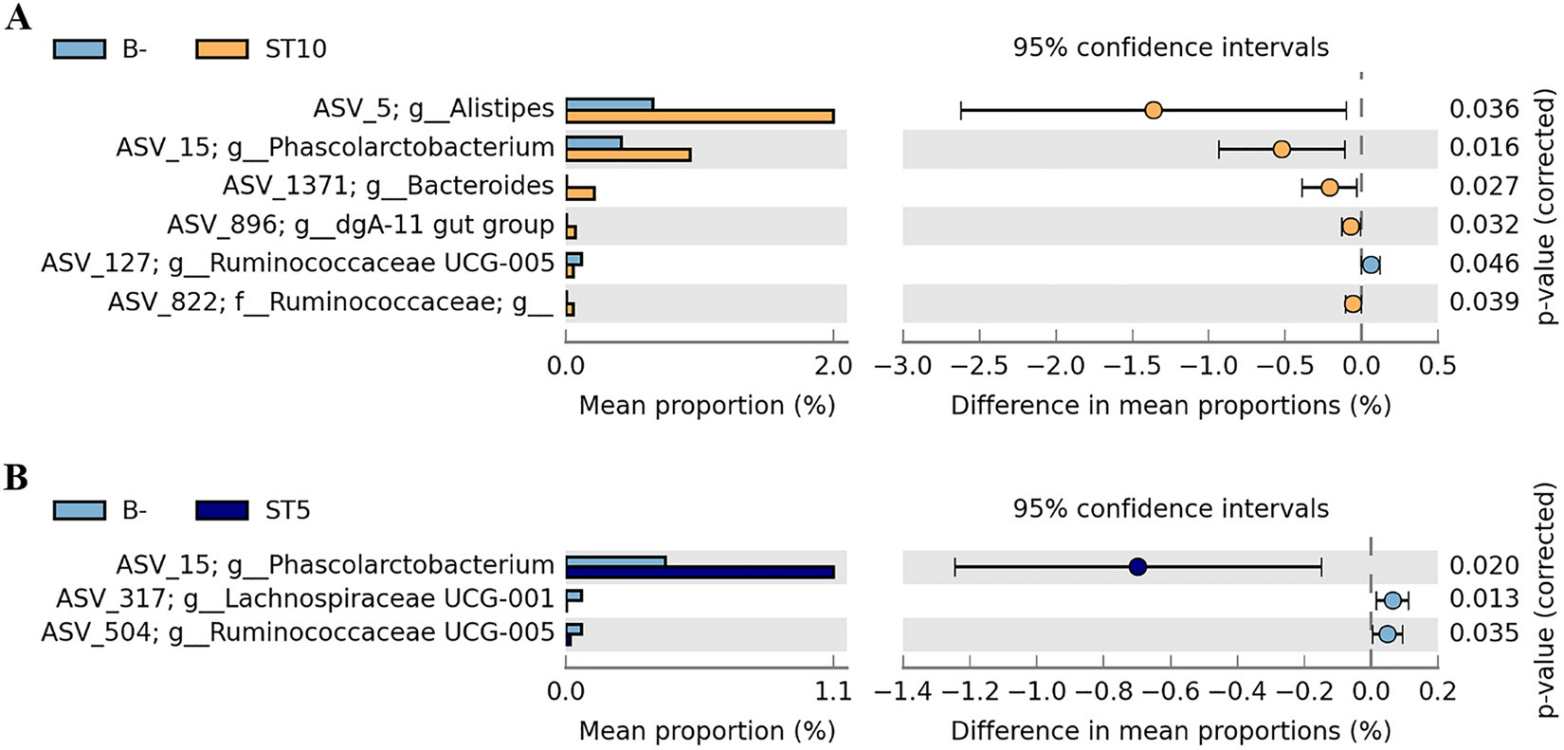
(A and B) Proportion of sequences assigned to each main group at the genus level, along with the means for each group and significance of difference in mean proportions using STAMP. Significant differences (*P* value < 0.05) are represented here between the two main groups (Welch’s *t* test, two-sided).

At the genus level, the heatmap of the 35 most abundant genera shows the similarities and differences among the three groups (Fig. S4 in the supplemental material). Furthermore, the taxonomic differences between the two groups were also compared using read counts normalized by DESeq2 in STAMP v 2.1.3 (30). The abundance of *Alistipes*, *Phascolarctobacterium*, *Bacteroides*, *Rikenellaceae*__dgA-11 gut group, and unclassified *Ruminococcaceae* in ST10-colonized forest musk deer were significantly higher than *Blastocystis*-free forest musk deer (Welch’s *t* test; all *P < *0.05), while the abundance of *Ruminococcaceae* UCG-005 was lower in ST10-colonized forest musk deer than in *Blastocystis*-free forest musk deer (*t *=* *1.014, *df *=* *1, *P = *0.046) (Fig. 5A). Similarly, higher abundance of *Phascolarctobacterium* were also observed in ST5-colonlized forest musk deer (*t *=* *3.112, *df *=* *1, *P = *0.02) (Fig. 5B). *Blastocystis*-free forest musk deer showed higher abundance of *Ruminococcaceae* UCG-005 and *Lachnospiraceae* UCG-001 when compared to ST5-colonlized forest musk deer (Welch’s *t* test; all *P < *0.05) (Fig. 5B).

**FIG 6:**
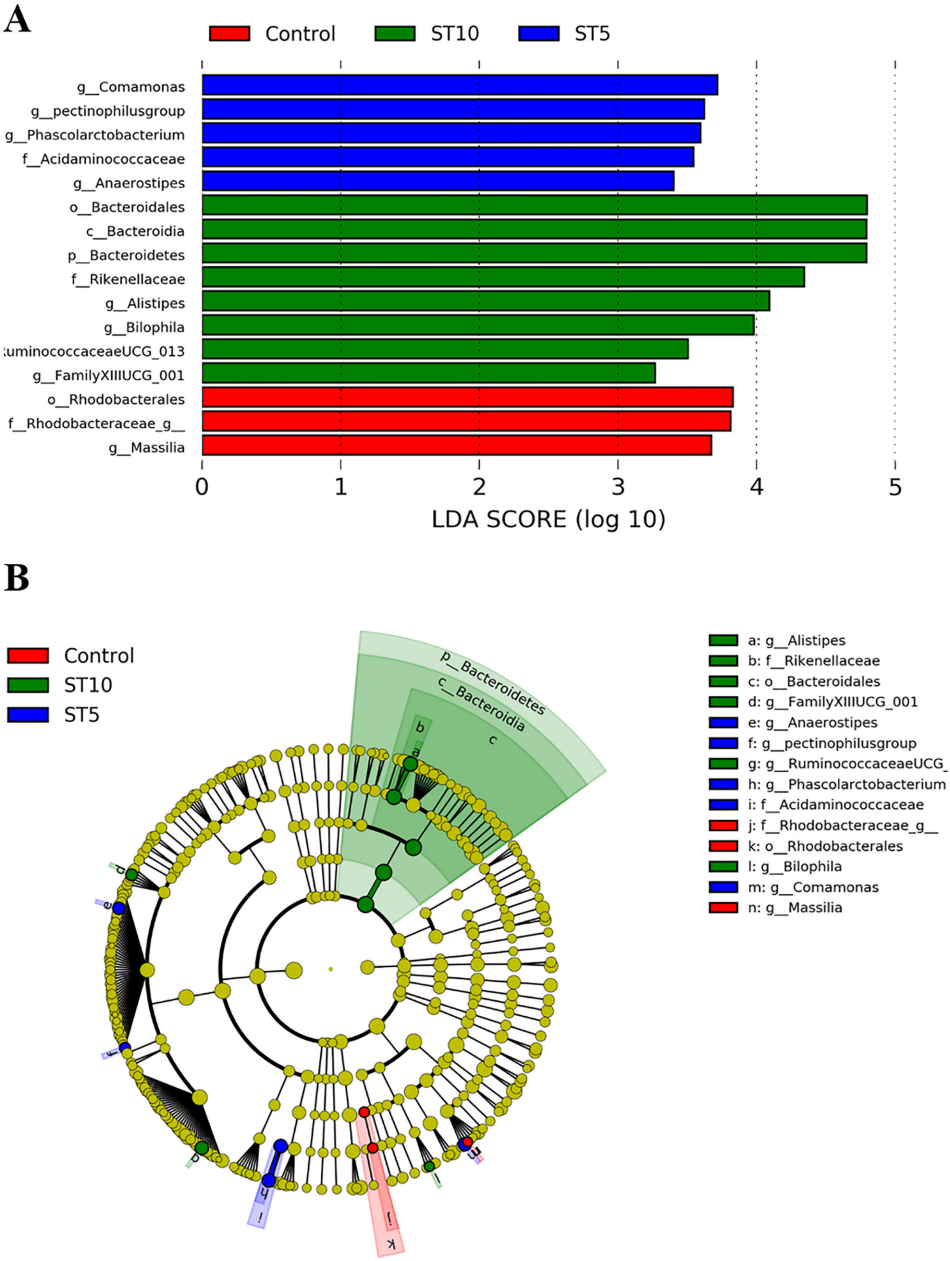
Differentially abundant bacterial taxa among the *Blastocystis*-free, ST10-colonized, ST5-colonized groups. Taxa with significantly differences in different groups were detected by LEfSe analysis (A). Cladogram generated by LEfSe indicating differentially abundant bacterial taxa (B).

Similarly, linear discriminant analysis effect size (LEfSe) analysis revealed that the *Bacteroidetes* (phylum), *Bacteroidia* (class), *Bacteroidales* (order), *Rikenellaceae* (family), *Alistipes*, *Bilophila*, *Ruminococcaceae* UCG-013, and *Clostridia* Family XIII UCG-001 (genus) were more abundant in ST10-colonized forest musk deer (Fig. 6). In contrast, *Acidaminococcaceae* (family), *Anaerostipes*, *Comamonas*, *pectinophilus* group, *Phascolarctobacterium* (genus) were enriched in ST5-colonzied forest musk deer. *Blastocystis*-free musk deer showed enrichment of *Rhodospirillales* (order), *Rhodobacteraceae* unclassified, and *Massilia* (genus) (all logarithmic LDA scores > 2, all *P < *0.05) (Fig. 6).

## DISCUSSION

*Blastocystis* is closely related to the health of the host and co-evolves with the host through complex interactions (31). Most studies have reported that the *Blastocystis* is associated with specific microbial profiles and bacterial taxa that are believed to be beneficial to intestinal and overall health in humans (32, 33). While only a few studies have studied the associations between *Blastocystis* and the intestinal microbiota of animal hosts. In wild chimpanzees, harboring similar gut microbiomes to humans, *Blastocystis* was associated with lower bacterial richness and higher abundance of *Enterobacteriaceae* and *Methanobacteriaceae* (34). Betts et al. showed that there was no significant difference in bacterial diversity and community compositions between water voles with and without *Blastocystis* (35). Our findings showed that *Blastocystis* ST10, but not ST5, is significantly associated with higher bacterial diversity and has a pronounced effect on microbial communities in forest musk deer.

Bacterial diversity is considered an important indicator of intestinal health, and high bacterial diversity implies stability and resilience of the gut ecosystem (36). Reduced microbial diversity has been observed in various chronic intestinal inflammation diseases, such as IBD (37). It has been determined that *Blastocystis* colonization is associated with increased fecal bacterial diversity in most of human microbiome studies, and thus *Blastocystis* is also considered to be a commensal or beneficial organism in humans (17, 38). A recent study also showed a greater bacterial richness in school-age children with *Blastocystis* colonization (39). Similar to human studies, ST10 colonization is associated with higher bacterial richness and diversity. However, it is worth noting that the present study was carried out with a relatively small sample size, and future studies will need larger sample sizes to confirm statistically significant associations.

Several microbiome studies have revealed *Firmicutes* and *Bacteroidetes* are the predominant gut bacterial communities of forest musk deer, although their gut microbiota is susceptible to seasonal variations (40, 41). Similarly, we found that, regardless of the presence or absence of *Blastocystis*, *Bacteroidetes*, and *Firmicutes* are also the two dominant phyla in the majority of the forest musk deer. A previous study has shown significant higher abundance of *Firmicutes* in the gut of wild forest musk deer compared to captive forest musk deer, while the abundance of *Bacteroidetes* in the gut of wild forest musk deer was significantly lower than in captive forest musk deer, suggesting different feeding and management methods could affect the gut microbiota of forest musk deer (26). Interestingly, we observed ST10-colonized forest musk deer had higher abundance of *Bacteroidetes* compared to *Blastocystis*-free forest musk deer. Bacteria from the phylum *Bacteroidetes* contribute to degrade carbohydrates, especially polysaccharides, proteins, and other substances to improve the host's nutrient utilization (42). This suggests that ST10 colonization may exert beneficial effects on the intestinal health of forest musk deer.

Bacterial community comparisons indicated enrichment of *Alistipes*, *Phascolarctobacterium*, *Bacteroides*, *Rikenellaceae*__dgA-11 gut group, and *Ruminococcaceae* were strongly associated with the presence of *Blastocystis* ST10. *Alistipes* are anaerobic bacteria commonly found in the gastrointestinal tract of healthy individuals with beneficial effects on host health (43). Accumulating evidence indicate that *Alistipes* may have protective effects against some diseases, such as Crohn’s disease, and colorectal cancer (44). The enrichment of *Alistipes*, together with anti-inflammatory cytokine IL-10 and regulatory T cells, could attenuate severe colitis in NOD2 knockout mice (45). *Alistipes*, *Bacteroides*, and *Rikenellaceae*__dgA-11 gut group belong to *Bacteroidetes*, which, in mouse models, has been shown to improve lipid metabolism by regulating acetic acid production (46, 47). Bacteria belonging to *Phascolarctobacterium* and *Ruminococcaceae* are known short chain fat acids (SCFAs) producers, especially for acetate, butyrate, and propionate, which can reduce intestinal inflammation and maintain the gut homeostasis (48, 49). Similarly, ST5-colonized forest musk deer also showed the enrichment of *Phascolarctobacterium*, belonging to *Acidaminococcaceae* family. Besides, the genera *Anaerostipes* and *pectinophilus* group, belonging to *Lachnospiraceae* family, were also more abundant in ST5-colonized group. The members of *Lachnospiraceae*, one of the major taxonomic groups of the gut microbiota, were able to degrade complex polysaccharides to SCFAs (50). The elevated abundance of *Lachnospiraceae* also showed the anti-inflammation effects in obese mice (51). Higher levels of *Comamonas*, a genus of *Proteobacteria*, were found in ST5-colonized forest musk deer. *Comamonas* species are common environmental bacteria occasionally associated with human infections (52). Current studies have shown that bacteria from genus *Comamonas* can cause appendicitis and bacteremia (53, 54). As the small number of ST5-positive samples included in this study, it is uncertain whether the effect of ST5 colonization on the gut microbiota of the forest musk deer is beneficial or not. Therefore, future studies should include more ST5-positive samples to further confirm these effects on the gut microbiota.

In this study, we observed ST10 colonization was associated with pronounced effects on gut microbiota, while ST5 did not show such influences on gut microbiota. Indeed, it has been determined that different subtypes (ST1, ST4, and ST7) exhibited considerable variation in the number of protein-coding genes (55). The proteases encoded by these genes are considered to be potential virulence factors of *Blastocystis*, and are important components involved in many essential biological processes (56). This could provide a possible explanation for the variable influences of *Blastocystis* on gut microbiota. Similar observations have been reported in for other *Blastocystis* subtypes. For example, *Blastocystis* ST4 colonization is associated with higher bacterial richness and high proportion of bacterial genera *Sporolactobacillus* and *Candidatus carsonella*, while ST3 did not show such significant relationships (57). Similarly, ST4 colonization is positively associated with the proportion of beneficial bacteria, *Akkermansia* and *Methanobrevibacter*, in healthy individuals in Europe, while ST3 colonization showed the reverse correlations (16). These results further confirm that different subtypes may have distinct functional potential for the microbiota profiles in the gut of host.

In summary, the present study characterized, for the first time, the associations between *Blastocystis* and gut bacterial community of forest musk deer. We observed the intersubtype variability (ST5 and ST10) for *Blastocystis*-microbiota interactions, which is mainly reflected in the difference in bacterial diversity and community composition. The presence of ST10 was associated with a higher bacterial diversity and the proportion of beneficial bacteria, such as *Alistipes* and *Bacteroides*, in the gut of forest musk deer, while ST5 colonization does not seem to be related to the specific gut microbial profiles of forest musk deer. These results suggest that different subtypes are differentially associated with the host intestinal microbiota. Further research should be aimed at elucidating the mechanism by which *Blastocystis* modulates the intestinal bacterial community and determining the contributions of specific bacterial taxa to the physiology of the forest musk deer.

## MATERIALS AND METHODS

### Sample collection.

Between August and September 2020, a total of 504 fecal samples were collected from captive forest musk deer in Sichuan province, southwestern of China. Fecal samples were collected from four captive breeding farms, 139 samples in Shimian (29°16' N, 102°20' E) at an altitude of 2,572 m, 144 in Hanyuan (29°29' N, 102°37' E) at an altitude of 1,076 m, 131 in Dujiangyan (31°01' N, 103°35' E) at an altitude of 739 m, and 90 in Maerkang (31°53' N, 102°07' E) at an altitude of 2,526 m. The forest musk deer breeding farms were cleaned the night before sampling, and each individual was kept in a separate enclosure so that the fresh feces of each individual could be collected the following morning. Feeding forms were similar across the four breeding farms, consisting mainly of some fresh leaves, carrots, and asparagus lettuces, etc. Approximately 5–10 g fresh fecal samples were collected using sterile disposal latex gloves and then put into separate sterile self-sealed bags to avoid sample cross-contamination. All samples were immediately transported to the laboratory and later stored at −80°C until processing. All animals were healthy, and none showed any clinical signs of gastrointestinal disease at the time of sampling.

### DNA extraction and *Blastocystis* identification.

Total genomic DNA was extracted using QIAamp Fast DNA Stool minikit (Qiagen, Germany) according to the manufacturer’s instructions. The extracted DNA were firstly screened for the presence of *Blastocystis* by PCR (PCR) amplification of the barcode region of the *SSU* rRNA gene, using primers RD5 (5′-ATCTGGTTGATCCTGCCAGT-3′) and BhRDr (5′-GAGCTTTTTAACTGCAACAACG-3′) (58). The PCR mixture (25 μL) contained 12.5 μL *Taq* PCR Master Mix (Sangon Biotech Co., Ltd., Shanghai, China), 1 μL each primer (0.4 μM), 2 μL genomic DNA, 1.5 mM MgCl_2_, and nuclease-free water up to desired volume. The PCR program includes 94°C for 4 min, followed by 30 cycles of 95°C for 15 s, 60°C for 15 s, and 72°C for 30 s, with an extension at 72°C for 5 min. Positive and negative controls were included in all the PCR tests. PCR products were subjected to 1.5% agarose gel (AddGene, Watertown, MA) electrophoresis and visualized by staining with SYBR Safe DNA Gel Stain (Thermo Fisher Scientific). PCR products with expected fragments (around 600 bp) were subsequently cleaned up using the QIAquick PCR Purifcation Kit according to the manufacturer’s instructions (Qiagen) and sent for sequencing (Sangon Biotech). Raw sequencing data were checked using Chromas 2.6.6 software to guarantee the accuracy of nucleotides. Trim ambiguous bases at the beginning and end of the sequences, and then the trimmed sequences were subjected to BLAST searches (http://blast.ncbi.nlm.nih.gov/Blast.cgi) to identify the *Blastocystis* subtypes. Two subtypes (ST5 and ST10) were identified, and both collected from Dujiangyan and Maerkang farms (see Table S1 in the supplemental material). To avoid the influence of age and gender differences on the microbiome results, we only included male forest musk deer of similar age (1.5–3 years old) for 16S rRNA gene sequencing. ST5 (OK445534) and ST10 (OK445535) showed 92.7% sequence similarities.

### 16S rRNA gene sequencing.

After *Blastocystis* identification, the *Blastocystis*-positive and *Blastocystis*-free samples (control) were used for the next 16S rRNA gene sequencing. Specifically, genomic DNA samples were purified using the Zymo Research BIOMICS DNA Microprep Kit (Cat# D4301) and its integrity was determined using 0.8% agarose electrophoresis. The nucleic acid concentration was detected using Tecan F200 detection (PicoGreen dye method). The V4 region of the 16S rRNA gene was amplified using the 515-F (5′-GTGYCAGCMGCCGCGGTAA-3′) and 806-R (5′-GGACTACHVGGGTWTCTAAT-3′) primers. Gene amplification was carried out using TOYOBO KOD-Plus-Neo DNA polymerase (KOD-401B). Single amplifications were performed in 50 μL reactions with 40 ng of template DNA. Cycling protocol consisted of 94°C for 1 min, followed by 30 cycles of 94°C for 20 s, 54°C for 30 s, and 72°C for 30 s, with final extension of 72°C for 5 min. PCR products were mixed with a 6-fold loading buffer, followed by electrophoresis of the target fragment using 2% agarose gel. The qualified samples were extracted from agarose gel using Zymoclean Gel Recovery Kit (D4008). Quantitative of samples were detected using Qubit @2.0 Fluorometer (Thermo Scientific), and then mixed it in equal mole quantities. Sequencing libraries were generated using NEBNext Ultra DNA Library Pre Kit for Illumina (NEB#E7645L). Qualified library was sequenced on an Illumina PE250 platform using Hiseq Rapid SBS Kit v2 (FC-402-4023 500 Cycle).

### Bioinformatic and statistical analysis.

Paired-end reads were assigned to samples based on their unique barcodes and truncated by cutting off the barcode and primer sequences. Paired-end clean reads were merged using FLASH (59), and the splicing sequences were called tags. The tags were compared with the reference database using UCHIME algorithm to detect and remove chimera sequences (60), and then obtained effective tags. Quantitative Insights into Microbial Ecology (QIIME2 version 2021.02) was used to for quality control (61, 62). The quality control standards are as follows: i) Filter out sequences with average mass less than 25; ii) The sequences with length less than 200 bp were removed; iii) Remove sequences with fuzzy base (N) number greater than 2. Taxonomic assignment was performed using the BLAST fitted classifier trained on the SILVA 138 reference database with the feature-classifier plugin for QIIME2 (63) based on 100% similarity. Biodiversity index analysis was calculated using QIIME2 and displayed with R software. For community composition analysis, R language (Version R-4.0.3) was used for various data conversion and ggplot2 package mapping. For Alpha diversity analysis, Observed species, Pielou's evenness, and Shannon indexes were identified using Vegan package in R. Pairwise comparisons of microbial communities in different groups were carried out using permutational multivariate analysis of variance (PERMANOVA, Weighted Unifrac distance) implemented in *adonis* (64). Principle coordinate analysis (PCoA) and heatmap analysis was performed using R package.

### Statistical analysis.

Statistical analysis was performed using R-4.0.3 software. Significant differences in alpha diversity (Observed species, Pielou's evenness, and Shannon indexes) between groups were determined using Kruskal-Wallis test. The significance of the microbial difference between groups was assessed by the Wilcoxon rank-sum test and PERMANOVA. The microbial community was further analyzed by using the STAMP v2.1.3 (Welch’s *t* test, two-sided) to reveal differences between the groups. LEfSe analysis was performed to detect bacterial taxa with significantly different abundance among different groups with *P* value < 0.05 and LDA score > 2 (65). LEfSe analysis was performed on the website http://huttenhower.sph.harvard.edu/galaxy/. *P* values < 0.05 were considered statistically significant.

### Data availability.

The raw sequence data from the fecal microbiota in this paper were uploaded to the Sequence Read Archive (SRA) database at NCBI under BioProject ID PRJNA850511.
