# Supplementary material for: Changes in Gut Microbiota Composition Associated with the Presence of Enteric Protist Blastocystis in Captive Forest Musk Deer (Moschus Berezovskii)
Source: Microbiol Spectr. 2022 Jun 23;10(4):e02269-21. doi: 10.1128/spectrum.02269-21 (PMC9430526; doi:10.1128/spectrum.02269-21)
Supplement: Supplemental file 1 — Supplemental material. Download spectrum.02269-21-s0001.pdf, PDF file, 0.6 MB [file spectrum.02269-21-s0001.pdf]

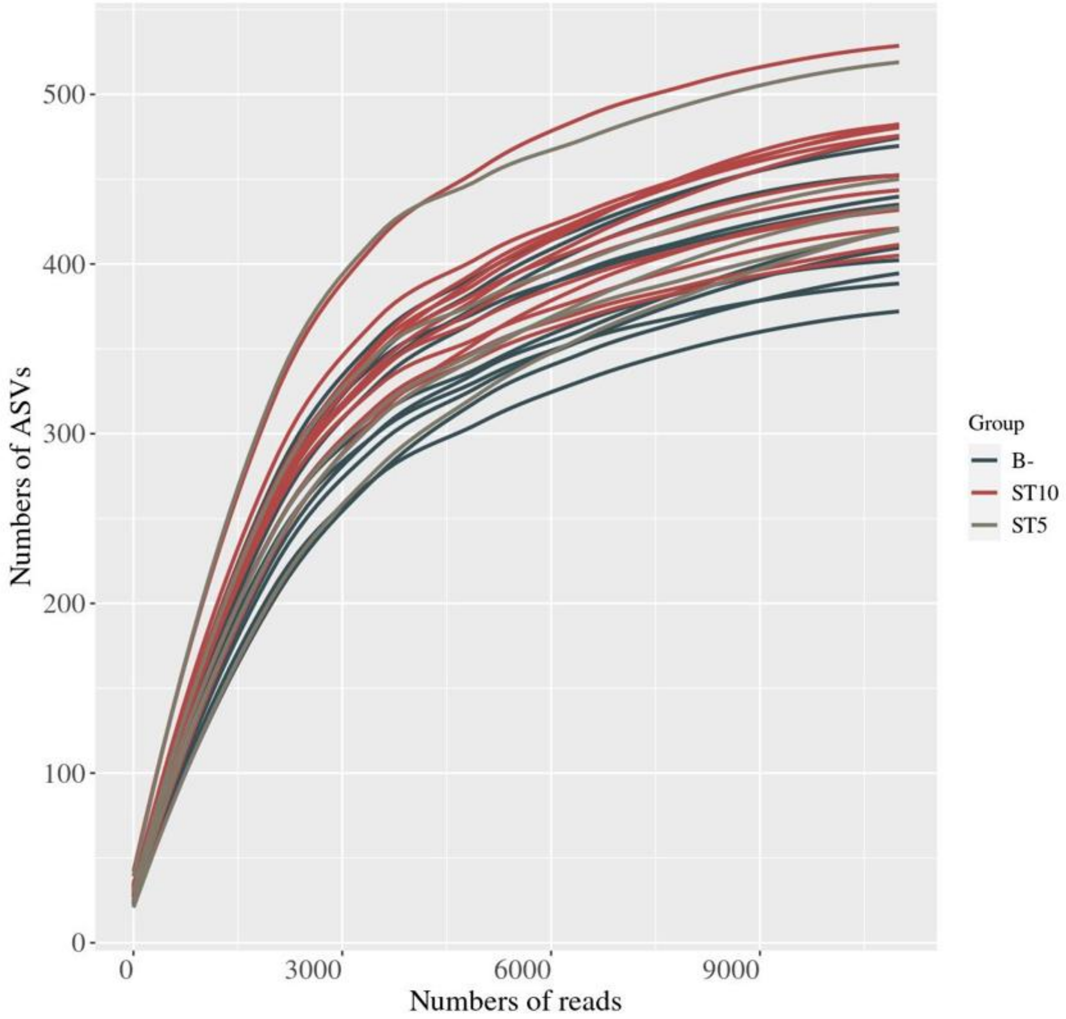

Figure S1: Rarefaction curves. The x-axis shows the number of reads per sample and the y-axis shows the observed species (amplicon sequence variants, ASVs). Each curve in the graph represents a different sample and the samples in the same group are represented by a uniform color. B-: *Blastocystis*-free forest musk deer. ST5: ST5-colonized forest musk deer.

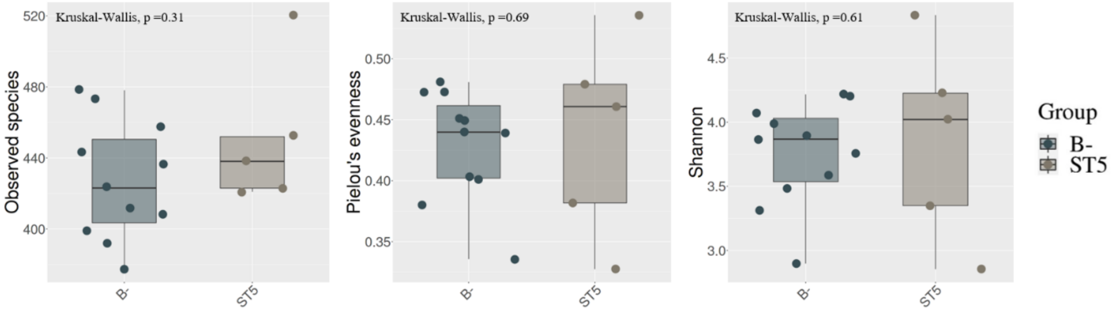

Figure S2: Comparison of alpha-diversity of gut microbiota from the ST5-colonized and *Blastocystis*-free groups based on Observed species, Pielou's evenness, and Shannon indexes.

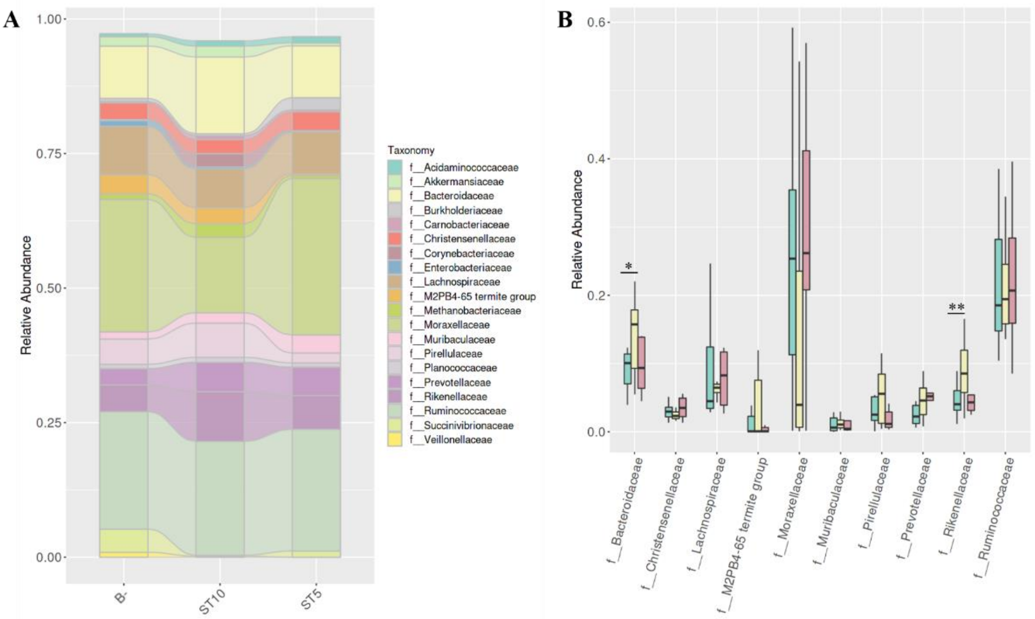

Figure S3: Relative abundance of the top 20 families (A). Relative abundance (mean ± SD) of 10 major bacterial families (B) *Blastocystis*-free, ST10-colonized, and ST5-colonized forest musk deer. Wilcoxon rank-sum test. $^{*}P < 0.05$, $^{**}P < 0.01$.

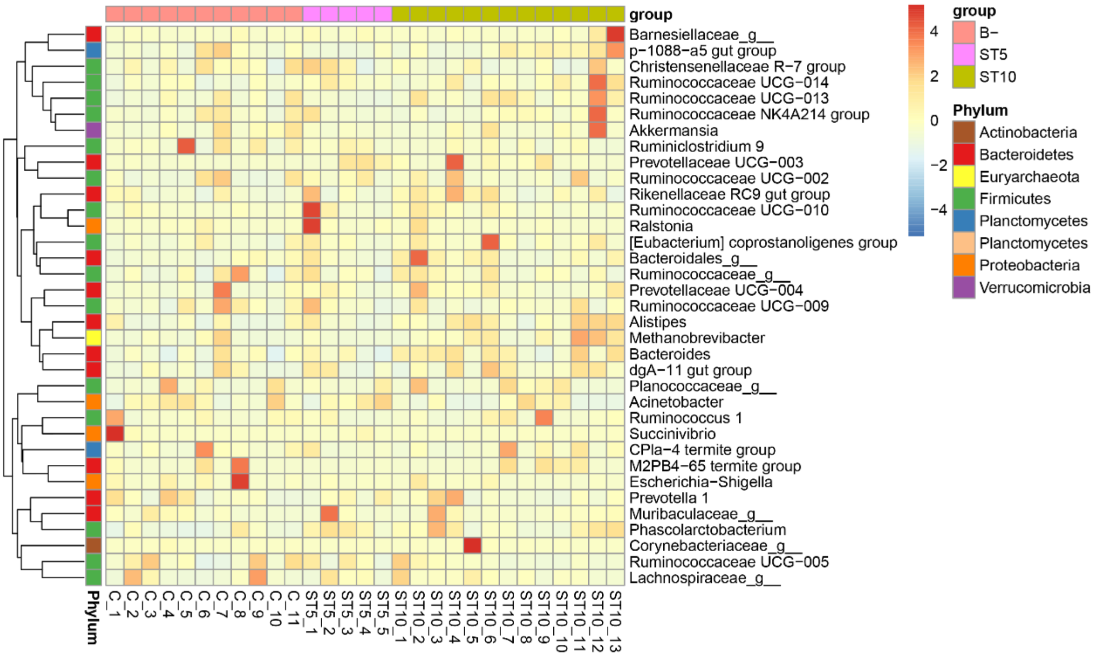

Figure S4. Heatmap of the 35 most abundant genera in *Blastocystis*-free, ST10-colonized, and ST5-colonized forest musk deer.

Table S1: The distributions of *Blastocystis*-positive samples.

Sample ID	Location	Sample ID	Location
C_1	Dujiangyan	ST10_1	Maerkang
C_2	Dujiangyan	ST10_2	Maerkang
C_3	Dujiangyan	ST10_3	Maerkang
C_4	Dujiangyan	ST10_4	Maerkang
C_5	Dujiangyan	ST10_5	Maerkang
C_6	Dujiangyan	ST10_6	Maerkang
C_7	Maerkang	ST10_7	Maerkang
C_8	Maerkang	ST10_8	Dujiangyan
C_9	Maerkang	ST10_9	Dujiangyan
C_10	Maerkang	ST10_10	Dujiangyan
C_11	Maerkang	ST10_11	Dujiangyan
ST5_1	Dujiangyan	ST10_12	Dujiangyan
ST5_2	Dujiangyan	ST10_13	Dujiangyan
ST5_3	Dujiangyan		
ST5_4	Dujiangyan		
ST5_5	Dujiangyan		
